# Body image dissatisfaction, depression, and anxiety in adolescents with celiac disease

**DOI:** 10.3389/fped.2025.1603009

**Published:** 2025-07-17

**Authors:** Buket Daldaban Sarıca, Esra Demirci, Derya Altay, Duran Arslan

**Affiliations:** ^1^ Department of Pediatric Gastroenterology, Hepatology, and Nutrition, Kayseri City Training and Research Hospital, Kayseri, Türkiye; ^2^ Department of Child and Adolescent Psychiatry, School of Medicine, Erciyes University, Kayseri, Türkiye; ^3^ Department of Pediatric Gastroenterology, Hepatology, and Nutrition, School of Medicine, Erciyes University, Kayseri, Türkiye

**Keywords:** celiac disease, body image, depression, anxiety, children

## Abstract

**Introduction:**

Celiac disease, also known as gluten-sensitive enteropathy, is an immune-mediated disorder with a broad clinical manifestations including psychiatric symptoms. The present study aims to evaluate body image dissatisfaction, depression, and anxiety among adolescents with celiac disease and to draw attention to the diagnosis and management of these comorbidities.

**Methods:**

This study was performed in Pediatric Gastroenterology unit of Erciyes University between July 2022 and June 2023. Adolescents aged 12–18 years diagnosed with histopathologically confirmed celiac disease and their age- and sex-matched healthy peers, were recruited in the study.

**Results:**

A significant difference was found between the patient and control groups in depression (*p* < 0.001), and body image dissatisfaction (*p* < 0.001). Adolescents who were non-compliant with the diet showed significantly elevated risk of body image dissatisfaction and depression compared with participants who adhered to the strict diet (*p* = 0.001, *p* < 0.001, respectively). A positive correlation was observed between tTGA-IgA antibody levels and depression (*r* = 0.618; *p* < 0.01), and a negative correlation was found between tTGA-IgA antibody levels and body image dissatisfaction scores (*r* = −0.400, *p* = 0.014).

**Conclusion:**

The present results underscore adolescents with celiac disease are at an heightened risk for psychiatric burden. Therefore, periodic follow-up should be performed to determine body image satisfaction in adolescents with celiac disease and to recognize mood-related symptoms. Early identification of symptoms associated with mood disorders and body image dissatisfaction in adolescents is critical for efficient patient care within celiac disease and to enhance adolescents’ holistic well-being.

## 1 Introduction

Celiac disease (CD) is a prevalent autoimmune disorder of the small intestine precipitated by an immune response to gluten (wheat, rye, and barley) in genetically susceptible individuals (1). CD is typically manifests in the pediatric population with diarrhea, abdominal bloating, and failure to thrive, whereas in older children it more often presents with bloating, constipation, or weight loss (2). Extraintestinal symptoms are frequent in both children and adolescents. A wide range of neurological sequelae may also occur in patients with CD, encompassing neuroinflammation, anxiety, recurrent headaches, and depressive features (3).

Depression and anxiety are among the most common emotional disorders in adolescents (4). Depression affects approximately 1.4% of adolescents aged 10–14, rising to over 3% by age 19 (5).

Anxiety disorders impact 4.4% of youths aged 10–14 and 5.5% of those aged 15–19 (5). Depressive disorders and anxiety can significantly disrupt school attendance and educational performance (6). Social withdrawal can deepen both disconnection and loneliness (7). Chronic illnesses, such as celiac disease, can precipitate the development and worsening of psychiatric conditions (8). Research on adult CD cohorts has documanted higher rates of depressive symptoms and anxiety, yet adolescentfocused data is limited (9). Pediatric patients with CD are more susceptible to depression and anxiety than their healthy peers, with observed rates of 3.5% and 3.7%, respectively (9). One investigation reported that nearly 40% of children with CD exhibited clinically significant levels of depressive and anxious symptomatology (8). Body image encompasses the thoughts, perceptions and feelings individuals hold about their physical form and the experiential aspects of embodiment (10). It has been estimated that body image disturbances affect 1.7%–37% of adolescents (10). In particular, these disturbances are often linked to mood disorders, pointing to a potantial overlap of psychological distress and body image dissatisfaction (BID) (10). Accordingly, it is reasonable to predict that body image issues will be a markedly prominent among adolescents with CD. Thus far, no study has specifically examined body image dissatisfaction in an adolescent cohort with CD, making this the first study the first of its kind.

Our clinical experience suggest a link between BID and depression in children with CD; nevertheless, data exploring the association between pediatric CD, body image concerns, and psychiatric symptoms remain sparse. These findings underscore the critical importance of early screening and management of these comorbid conditions. Given the complexity of CD and the distinct challenges posed by adolescence, integrated approach is vital to address the psychiatric comorbidities in this population.

## 2 Materials and methods

### 2.1 Study design

We performed a survey-based prospective cohort study on adolescents diagnosed with CD followed up at Erciyes University Department of Pediatric Gastroenterology, between July 1, 2022 and June 15, 2023. The informations of children were reviewed form the hospital clinical records, and children and/or their parents provided written informed consent to enroll in the study. Questionnaire responses were collected prospectively during follow-up visits. The study was ethically approved by the local committee (2022/459).

### 2.2 Participants, data collection and questionnaires

This study included adolescents who were diagnosed with CD based on positive serological markers (Anti-tissue Transglutaminase IgA antibody) and confirmation through duodenal biopsies, in accordance with the European Society for Paediatric Gastroenterology, Hepatology and Nutrition (ESPGHAN) guidelines (11).

The control group consisted of healthy children with no chronic disease. Clinical and laboratory data were retrieved from hospital records. All participants filled out a series of standardized questionnaires, and the total scores were methodically recorded for analysis.

The Body Perception Scale consists of 40 items, each rated on a 5-point Likert scale, ranging from 1 ‘did not like at all’ to 5 ‘liked very much’. The total score ranges from 40 to 200, with higher scores indicating a more positive perception of one's body image (12). The Kovacs Depression Inventory is a validated instrument used to assess cognitive, behavioral, and emotional symptoms of depression in adolescents (13). It consists of 27 items addressing aspects such as depressed mood, hopelessness, and impaired social functioning. The total score ranges from 0 to 54, with the following interpretation: 0–8: No indication of depression; 9–19: Subthreshold depressive symptoms; ≥20: Indicative of clinical depression. Elevated scores correspond to greater severity of depressive symptoms. The Childhood Anxiety Sensitivity Index includes 18 items evaluating children's emotional responses to anxietyinducing stimuli (14). Each item is scored as 1 ‘not at all’, 2 ‘a little’, or 3 ‘a lot’, yielding a total score range of 18–54. Higher scores indicate increased sensitivity to anxiety-related sensations (15).

### 2.3 Statistical analysis

All data were analyzed using Statistical Package for the Social Sciences (SPSS), version 22.0 for Windows. Descriptive statistics [counts, percentages, means, standard deviations, medians and interquartile ranges (IQR)] were used. Chi-square test was used for categorical variables in comparisons between groups. For continuous variables that were not normally distributed, Mann–Whitney *U* test was applied to two-group comparisons and Kruskal–Wallis H test to three-group comparisons. When Kruskal–Wallis test showed a significant difference, pairwise group comparisons were used and the direction of the difference was indicated. Associations between continuous variables were evaluated using Spearman's rank correlation.

## 3 Results

### 3.1 Participant characteristics

A total of thirty-seven patients with CD were included, with a median age of 14.4 (IQR = 2.5) years; 64.9% were female. Eight patients (21.6%) had additional chronic diseases, including Type 1 Diabetes Mellitus (*n* = 4) (others: eosinophilic esophagitis, Hashimoto thyroiditis, vitiligo, and juvenile idiopathic arthritis). Of these patients, 64.9% reported adherence to the gluten-free diet (GFD), while 35.1% were non-adherent. The mean duration since diagnosis of CD was 3.92 ± 2.59 years, and the mean antibody level [tissue Transglutaminase IgA (tTG-IgA)] was 228.95 ± 55.64 U/ml.

According to the Mann–Whitney *U* test, significant differences were found between groups in weight SDS (*p* = 0.002), depression (*p* < 0.001), and body image (*p* < 0.001). Descriptive data for the groups are presented in Table 1.

**Table 1:** Descriptive profile of the CD and control groups.

Descriptives		CD		Control		*p*
		*n*	%	*n*	%	
Gender	M	13	35.1	14	37.8	*χ*^2^ = 0.058
	F	24	64.9	23	62.2	*p* = 0.500
Diet compliance	No	13	35.1			
	Yes	24	64.9			
Complaint	Abdominal pain	7	18.9			
	Inadequate weight gain	10	27.0			
	Short stature	10	27.0			
	Persistent diarrhea	2	5.4			
	Weakness	2	5.4			
	Family history of CD	6	16.2			
		Median	(IQR)	Median	(IQR)	MWU/p
Age		14.44	2.5	15.0	2.0	571.5/0.215
Weight SDS		−1.15	1.8	−0.29	1.6	402.0/0.002
Height SDS		−0.50	2.1	−0.20	1.58	546.5/0.136
Body Image score		131	36	154	48	354.5/0.000
Depression score		17	24	2	4	225.5/0.000
Anxiety score		31	9	31	7	630.0/0.555

Chi-Square Analysis; Mann–Whitney *U* Test.

### 3.2 Clinical outcomes

When body image, depression, and anxiety scores in CD group were examined by demographic characteristics, no significant differences found based on gender. Among patients who did not adhere to the GFD, the median depression score was 29.0 (IQR = 24), compared to 8.0 (IQR = 16) for those who followed it, indicating a significant difference (*p* < 0.001). Corresponding body image scores were 113.0 (IQR = 38) for those who did not follow the GFD and 140.5 (IQR = 31) for those who did, indicating a significant difference (*p* = 0.001). In contrast, no significant difference was observed in anxiety levels (*p* = 0.089) (Table 2). No significant difference was found between patients with and without additional chronic diseases in terms of depression, anxiety, and BID.

**Table 2:** Differences in body image, depression, and anxiety scores according to descriptive characteristics.

Descriptives	*n*	Body image	Depression	Anxiety
Gender		Median (IQR)	Median (IQR)	Median (IQR)
M	13	132.0 (23)	13.0 (19)	31.0 (9)
F	24	129.5 (55)	17.0 (26)	34.0 (9)
MWU=		154.0	154.0	144.0
*p*=		0.949	0.949	0.702
Diet compliance		Median (IQR)	Median (IQR)	Median (IQR)
No	13	113.0 (38)	29.0 (24)	35.0 (9)
Yes	24	140.5 (31)	8.0 (16)	29.0 (10)
MWU=		59.5	45.5	102.5
*p*=		0.001	0.000	0.089
		Body Image (r/p)	Depression (r/p)	Anxiety (r/p)
Age		0.115/0.499	0.194/0.250	0.2181/0.284
Weight SDS		0.294/0.078	0.134/0.431	0.187/0.269
Height SDS		0.215/0.201	0.010/0.952	0.232/0.168

Mann–Whitney *U* test; Spearman's rank correlation.

Table 3 presents group-based differences in body image, depression, and anxiety scores. A Kruskall–Wallis H test revealed significant differences in both depression and body image scores. The highest median depression score was observed in the GFD non-adherent group, followed by the adherent group and the control group, yielding a statistically significant difference (KWH = 32.941, *p* < 0.001). For body image scores, the control group recorded the highest score, followed by the adherent and the non-adherent group, again indicating a significant difference (KWH = 19.889, *p* < 0.001).

**Table 3:** Differentiation Status of scores according to groups.

Study measures	GFD non-adherent^1^		GFD Adherent^2^		Control^3^		KWH	*p* difference
	Median	IQR	Median	IQR	Median	IQR		
Depression	29.0	24	8.0	16	2.0	4	32.941	0.000 1 > 2,3; 2 > 3
Anxiety	35.0	9	29.0	10	31.0	7	3.278	0.194
Body Image	113.0	38	140.5	31	154.0	48	19.889	0.000 2.3 > 1; 3 > 2

Kruskall–Wallis H test.

### 3.3 Correlation analyses

As shown in Table 4, there is a positive correlation between depression and anxiety (*r* = 0.388; *p* = 0.018). In contrast, a significant negative association was found between depression and body image scores (*r* = −0.641; *p* < 0.01), indicating that higher levels of depression correspond to more negative body image satisfaction. The positive correlation between depression and disease duration (*r* = 0.158; *p* = 0.350) was not statistically significant. However, a strong, significant positive correlation detected between antibody levels and depression (*r* = 0.618; *p* < 0.01) and a negative correlation detected between antibody levels and body image scores (*r* = −0.400, *p* = 0.014), suggesting that higher antibody levels are linked to increased depression and decreased body image scores.

**Table 4:** Correlation analysis.

Study measures	*r/p*	Depression	Anxiety	Body image	Disease duration	tTG-IgA
Depression	*r*	1.000				
	*p*	0.000				
Anxiety	*r*	0.388*	1.000			
	*p*	0.018	0.000			
Body Image	*r*	−0.641**	−0.357*	1.000		
	*p*	0.000	0.030	0.000		
Disease Duration	*r*	0.158	0.013	−0.084	1.000	
	*p*	0.350	0.939	0.620	0.000	
tTG-IgA	*r*	0.618**	0.122	−0.400*	0.066	1.000
	*p*	0.000	0.471	0.014	0.700	0.000

tTG-IgA, tissue transglutaminase IgA.

* <0.05; **<0.01; Spearman's rank correlation.

## 4 Discussion

This is the first study notably examining BID, depression and anxiety in adolescents with CD using a confirmed screening tool. Although CD was classically considered to be a gastrointestinal disorder mainly related to malabsorption, it is now more accurately classified as an autoimmune disease with systemic manifestations (16). The pathophysiological mechanisms behind neuropsychiatric aspects of CD remain controversial. Increased intestinal permeability allows the entry of molecules such as biomolecular aggregates, gliadin antigen, inflammatory mediators, and tissue transglutaminase (tTG)IgA antibodies into the circulation, contributing to the initiation or exacerbation of symptoms through production of the cross-reactive antibodies, direct toxicity and immune complex deposition in central nervous system (17). Additionally, molecular mimicry between gliadin and neural pathway proteins has been proposed to play a crucial role in the psychiatric manifestations of CD; however, the precise mechanism remains unclear (18). The most common psychiatric manifestations in CD are depression, anxiety, irritability, attention deficit hyperactivity disorder, and sleep complaints (19). Although studies have been conducted on depression and anxiety in CD (8, 20), its effects on adolescents are still worth investigating due to the limited available data and the significant impact of the negative consequences of the disease.

When individuals hold negative perceptions of their bodies or identify a difference between their ideal and actual physical characteristics, BID is present (21). Factors associated with BID include body mass index, social media exposure, peer pressure, sociocultural appearance norms, biological and psychological changes during adolescence, and chronic diseases that may influence physical appearance (22). The literature indicates that patients with chronic diseases have higher levels of BID compared to healthy controls (23). Although available data are limited, an association between BID and autoimmune diseases has been indicated in adults (24, 25). Studies investigating the relationship between GFD adherence and body image disturbances in adults with CD have reported conflicting results (26, 27). CD can lead to physical symptoms that adversely impact appearance, such as abdominal bloating/distension, underweight, and various skin problems such as dermatitis herpetiformis (16). In our study, BID was more prevalent in adolescents with CD compared to their healthy peers. In line with the literature, we suggest that the more unfavorable body perceptions seen in our patients may be associated with the negative physical consequences of CD.

Correlational studies have supported the link between food addiction, media exposure, and BID in adolescents. The most widely accepted theories that explain how media influences diet and body image are sociocultural and objectification theories. A key component of these theories is body surveillance, which may contribute to anxiety, eating disorders, and BID in adolescents (22). CD requires lifelong adherence to a strict GFD, which may lead to feelings of social exclusion during meals, fear of making dietary mistakes, and perceptions of insecurity or inadequacy regarding one's body. This psychological burden may contribute to increased BID, particularly in adolescents. Additionally, constant reading of food labels, food refusal, and continuous monitoring of eating behaviors may lead to obsessive body awareness in this age group (28). Gluten exposure is known to trigger not only gastrointestinal symptoms but also psychiatric manifestations such as, fatigue, brain fog, and irritability (16). We propose that these symptoms may indirectly contribute to BID by affecting sensations of bloating, illness, and a lack of control over the body. Repeated gluten exposure may also disrupt eating behaviors and increase food-related anxiety.

In CD, increased intestinal permeability results from the impairment of the zonulin–tight junction relationship, epithelial damage, and villous atrophy (29). The ongoing inflammatory response due to barrier dysfunction is manifested by persistent serum CD antibody levels. In our study, we observed that both BID and depressive symptoms were more prominent in patients with elevated serum antitTG-IgA levels. It can be hypothesized that gluten, through its effect of both physical symptoms and gut barrier impairment, may also aggravate mental and psychological symptoms via the gut–brain axis. Therefore, we suggest that the elevated anti-tTG-IgA levels detected in our study may help explain the relationship with BID. Furthermore, we observed that dietary non-compliance was associated with more adverse body image perception. However, body image satisfaction was higher among patients who adhered to the GFD, similar to that observed in healthy adolescents. These findings suggest that eliminating gluten exposure may reduce both physical symptoms and mood disorders that negatively influence body image. Although previous studies have shown that BID is more prevalent in girls (10), we did not observe a gender difference. This divergence may be attributed to the limited sample size in our study.

The prolonged course of disease duration may adversely impact both psychological body image and well-being by leading to decreased quality of life, social isolation, and limitations in daily activities. These factors may facilitate the development of BID, particularly during adolescence. Moreover, many individuals with BID are sensitive to rejection and may hesitate to express their concerns for fear of being perceived as superficial. BID is a serious condition; affected adolescents often suffer significantly and may experience severe psychological distress, including suicidality (30, 31). In our study, however, we did not observe a significant association between extended disease duration and levels of depression, anxiety, or BID. Larger-scale research is needed to explore this relationship more comprehensively. The duration of untreated depression is the most important factor influencing both the severity of the disease and the likelihood of recovery. Active treatment reduces the duration of depressive episodes (32). The US Preventive Services Task Force recommends screening for a major depressive disorder in adolescents aged 12–18 years (33).

In our study, we found that non-compliant patients are more prone to depression and body image disturbances compared to GFD compliant patients with CD and healthy adolescents. At the same time, even in adolescents with CD who were compliant with GFD, this tendency was greater than in healthy ones. Gliadin increases zonulin-dependent intestinal paracellular permeability, irrespective of disease status (34). This mechanism may explain the higher prevalence of depression and body image disturbances even in patients adhering to a strict GFD. While many of the symptoms of CD improve with a strict GFD, fatigue and some neurological symptoms may persist for a prolonged period in certain patients (1).

There are limited studies investigating anxiety in adults with CD. Some reported greater anxiety in CD compared to controls (35), while others did not (36). In our study, we did not find any susceptibility to anxiety in adolescents with CD. The literature has shown a relationship between depression and BID in adolescents (37). The individual's negative perception of his/her own body, especially in adolescence and young adulthood, leads to psychological states such as decreased self-esteem, feelings of inadequacy, social withdrawal, and self-shame. This situation significantly increases the risk of depression over time. Although there was a strong correlation between BID and depression and anxiety in our study, a relationship was established between depression and anxiety. However, we did not apply a generic quality-of-life instruments, as the emotional subscale scores may offer only a relatively weak and indirect presentation of emotional functioning status and anxiety burden. Since data on the mental states of adolescents with CD are quite limited, more comprehensive studies are needed on this subject.

## 5 Limitations

This study has several limitations. The cross-sectional design prevented us from making causal inferences between variables. Although our study was conducted prospectively using a survey-based approach, the generalizability of the results is limited due to the small sample size. Additionally, the reliance on self-reported data may have introduced information bias. Furthermore, long-term effects could not be assessed due to the short follow-up period.

## 6 Conclusion

This study presents insights into the psychiatric manifestations of CD in adolescents. Body image dissatisfaction, depression, and anxiety are conditions that may occur in children with CD and can negatively affect quality of life. Dietary compliance was associated with reduced depressive symptoms and improved body image perception. Therefore, it is crucial to inform the families of adolescents with CD about potential psychiatric and body image vulnerabilities in order to facilitate adaptation to a GFD and improve overall well-being.

## Data Availability

The datasets presented in this study can be found in online repositories. The names of the repository/repositories and accession number(s) can be found in the article/Supplementary Material.
